# The Prevention of Puerperal Fever

**Published:** 1895-04-13

**Authors:** 


### Medical Progress and Hospital Clinics.

[*The Editor will be glad to receive offers of co-operation and contributions from members of the profession. All letters should be addressed to* The Editor, The Lodge, Porchester Square, London, W.]

### THE PREVENTION OF PUERPERAL FEVER.

A few weeks ago we drew attention to the fact that the definition of puerperal fever is so vague that there is but small security that all the deaths from that disease are registered under that title. We now wish to emphasise in the strongest possible way the fact that the present practice of the medical profession is far in arrear of present knowledge in regard to the treatment of women in their confinements, and to indicate in simple terms the means by which the ghastly massacre of women which now goes on could and should be prevented. Large as is even the registered death-rate from puerperal fever among the general population, it used to be still larger among the inmates of lying-in hospitals; so much so that, writing even so lately as 1871 Florence Nightingale said: "Unless, then, it can be clearly shown that these enormous death-rates can be abated, or that they are altogether inevitable, does not the whole evidence with regard to special lying-in hospitals lead but to one conclusion—viz., that they should be closed? Is there any conceivable amount of privation which would warrant such a step as bringing together a constant number of puerperal women into the same room, in buildings constructed and managed on the principles embodied in existing lying-in institutions?" And yet, as is pointed out in the *Practitioner*, from these same buildings at the present day, by rigid attention to all the details of the antiseptic method, puerperal fever has almost disappeared. It is impossible to disregard the broad fact that from the very institutions in which puerperal fever was most rampant it has been abolished by antiseptic precautions, while outside, among the general mass of the people, among whom these precautions are notoriously carried out in a most perfunctory manner, the mortality has actually increased within recent years. During the ten years from 1861 to 1870 the average mortality from puerperal fever was 1·58 per 1,000 births, while during the twenty years from 1871 to 1890 the average was 2·59, and this, notwithstanding the probability, to which we recently alluded, that many cases are returned under other headings owing to the discredit and disability which now attaches to a confession of having had to do with a case of this nature. It is impossible, then, to avoid the conclusion that the knowledge we possess in regard to the prevention of this disease is not being put into practice, and that outside our lying-in hospitals antiseptics are being but very imperfectly applied. Speaking of another matter, the Prince of Wales some time ago made the pertinent remark, "If these diseases are preventible, why are they not prevented?" And to puerperal fever this question applies with double force, for, if we are to believe those who have studied the matter most deeply, the infection is hand-borne, and can be prevented by cleanliness and antiseptics.

In the application of the antiseptic method to midwifery in general practice there are three main points to be considered: (1) The cleansing of the hands and instruments; (2) the cleansing of the vulva; (3) the application of antiseptic douches to the inner parts. The first two are absolutely essential in every case, but the third is not the practice at all institutions, nor does its necessity appear to be equal in every case. Instruments should be boiled. There is no other method by which the same degree of asepsis can be produced; but in default of boiling they should be thoroughly soaked and scrubbed in a solution of carbolic acid (1 in 20). The hands and forearms of the doctor and the nurse should be cleaned first by turpentine, then by warm soap and water, after which the hands should be soaked for a minute and the forearms bathed in solution of corrosive sublimate (1 to 1,000), and every time the genitals of the patient have to be touched, either by doctor or nurse, the hands should be soaked in this solution and go to their work unwiped. A basin of 1 in 1,000 solution of corrosive must stand always ready for this purpose, not only during labour but during the puerperium. The process of cleansing the vulva is carried out in practically the same manner at most of the hospitals, and consists of washing with warm soap and water, followed by thorough swabbing with cotton wool soaked in solution of corrosive sublimate (1 in 2,000). This should always be done as soon as labour pains commence, and during the course of labour the vulva and surrounding parts should be frequently wiped with cotton wool wetted with the antiseptic. During the puerperium also the vulva and external parts should be wiped with cotton wool wetted with the antiseptic lotion when the diapers are changed, and it should be recognised that, either during labour or afterwards, the first step in making an examination, using an instrument, or passing a catheter, should be the antiseptic cleansing of the vulva. The use of the internal douche is a matter regarding which there is some difference of opinion. At Queen Charlotte's Hospital the vagina is irrigated with corrosive sublimate solution (1 in 2,000) on the patient entering the labour ward, and a warm vaginal douche of the same strength is given when labour is completed. At the Rotunda Hospital, however, according to the *Practitioner*, a vaginal douche is not used, either before, during, or after labour, in uncomplicated cases, nor during the puerperium. Continental experience also agrees with this. It is clear, therefore, that the routine use of the douche in ordinary cases is not an essential part of antiseptic midwifery, and by this admission we at once take away all force from the objection so often raised, viz., that the details of the process are so complicated as to be unsuited for private practice. The fact is that what we have described are mere matters of cleanliness, such as would be adopted in the simplest surgical operation, and should be looked upon as obligatory in every confinement.

There is one incidental advantage in the enforcement of these rules—they will tend greatly to diminish the frequency of internal examinations. We believe that in some German schools the introduction of the finger is not allowed when once a normal presentation has been determined, and at the Rotunda not more than four examinations are allowed in the whole course of a normal labour. Wherever instrumental interference or the introduction of the hand is necessary, the use of the vaginal douche of warm solution of corrosive sublimate (1 in 2,000) is to be advised, and also whenever any septic taint is present.

If a lubricant is required, vaseline containing 1 part of corrosive sublimate in 1,000 is probably the best, but if the hand be taken direct from the lotion no lubricant is wanted in ordinary examinations. It should always be remembered that soap decomposes corrosive sublimate, and that it should be washed off the hands before they are introduced into the antiseptic.

It is sad to think of the loss of life which goes on year by year from want of attention to such little details as we have here described. This loss of life has been stopped where they have been attended to, and we think that the time is past when any medical man can properly excuse himself for neglecting them by the vague assertion that he does not believe in antiseptics. Midwifery should be dealt with as carefully as surgery, and if this is too much trouble it should be refused. During the year 1893 there were 960 deaths from puerperal fever in those towns alone which have adopted the Notification of Diseases Act. It is a shocking mortality, and should be stopped.

